# Cooling Effect of Green Space and Water on Urban Heat Island and the Perception of Residents: A Case Study of Xi’an City

**DOI:** 10.3390/ijerph192214880

**Published:** 2022-11-12

**Authors:** Rong Huang, Mei Yang, Guohua Lin, Xiaoyan Ma, Xuan Wang, Qian Huang, Tian Zhang

**Affiliations:** 1 School of Geography and Tourism, Shaanxi Normal University, Xi’an 710119, China; 2 Northwest Land and Resource Research Center, Shaanxi Normal University, Xi’an 710119, China

**Keywords:** urban heat island, cooling effect, green space, water landscape, resident perception, Xi’an City

## Abstract

The urban heat island (UHI) is a typical environmental problem that affects people’s health and restricts urban development. Understanding the cooling effect of ecological landscapes and residents’ perceptions of the cooling effect can help guide urban planning and mitigate environmental risk. This study analyzed the spatiotemporal evolution of UHI in the central area of Xi’an City in 1999, 2006, 2014, and 2019, and compared the cooling effect of green space and water among 13 urban parks in 2019. Furthermore, we investigated the constraining effect of landscape patterns on UHI and residents’ perceptions. Our results show that the area of moderate temperature region increased significantly in the past 20 years, and the UHI of old urban areas has been reduced. The UHI hot spots generally migrated to the northwest, and a shrinking–transferring–diffusing trend was observed across three periods (1999–2006, 2006–2014, 2014–2019). In addition, the cooling effect of parks increased with the proportion of water area, and the average cooling intensity and cooling amplitude were measured at 3.00 °C and 241.43 m, respectively. It was identified that a larger area, a longer circumference, and a more regular shape were more beneficial in reducing the urban thermal environment. Based on 325 questionnaires, we found that 73.23% of residents believed that the cooling effect of green space and water has become better in recent years, but less-educated people tended to be pessimistic about this. Among the residents, 79.08 and 40.92%, respectively, believed that the area and shape of the ecological landscape had an influence on the cooling effect. The comparison of remote sensing inversion results and questionnaire responses revealed that it is critical to incorporate residents’ perceptions into urban construction planning for heat risk prevention.

## 1. Introduction

In recent years, against the background of global warming and accelerated urbanization, the increase in urban temperature has significantly outpaced that of global temperature [1]. The urban heat island (UHI) caused by population aggregation and high-density construction, is among the typical ecological effects of urbanization [2]. That is, when a city develops to a certain scale, due to factors such as underlying surface changes, air pollution, and anthropogenic heat emissions, the temperature in the urban area will be significantly higher than in the suburbs [3], resulting in local climate change [4]. By altering the heat transfer between the ground surface and the atmosphere, UHI can increase the risk of water pollution, air pollution, urban waterlogging, and other phenomena, thus has become a major threat to urban security and residents’ health. The construction of urban ecological landscapes is one of the nature-based solutions (NbS) for dealing with urban thermal environment stress [5]. Clarifying the cooling effect of urban landscapes has become a prerequisite for addressing the risks of environmental change. Meanwhile, integrating urban residents’ actual perceptions of UHI and the landscape cooling effect into relevant studies, will provide practical guidance for urban landscape optimization and green infrastructure construction.

With a growing emphasis on improving human settlement quality and ensuring sustainable urban development, the research priority of the urban heat island effect is shifting from field monitoring [6] and quantitative simulation [7] to adaptation and mitigation [8]. In terms of study subjects, current research on the cooling effect of the urban landscape is mainly focused on evaluating the cooling effect [9], identifying the influencing factors [10], analyzing the mechanism [11], and developing optimization methods [12]. Previous studies have shown that increasing the ecological land area and optimizing land use patterns can effectively reduce UHI [13,14]. With regard to methods, as the characteristics of the research sample transition from patch to landscape, large-sample and multi-scale land surface temperature (LST) retrieval based on Landsat, SPOT, IKONOS, and other remote sensing images, as well as the identification of green space and water landscape have been widely studied [15]. In addition, the development of indicators to measure the cooling effect has made it possible to compare the cooling capacity of landscapes. At present, commonly used indicators include cooling intensity [16], cooling amplitude [17], and cooling efficiency [18], which can provide key information such as the difference in average temperature between the ecological landscape and surrounding areas and the cooling effect range. In recent years, there has been a greater focus on the perception of heat waves caused by UHI [19,20]. Relevant studies have primarily focused on health risks and adaptive behaviors, and research on population vulnerability [21,22] and adaptive capacity [23] influenced by UHI has also provided effective support for improving the quality of the urban settlement environment.

Nevertheless, there are still some deficiencies in the current research on UHI mitigation methods, and mechanisms of the urban heat island effect deserve further investigation. First, many studies still focus on analyzing the cooling effect of urban green space, whereas relatively less attention is paid to the cold island effect of urban water, and the comparison between the two needs to be better defined. Second, research findings that can guide urban planning with strong operability and easy implementation are scarce. Research on the cooling effect of land use patches from the perspective of landscape ecology deserves further exploration. Third, there is still a lack of comprehensive discussion on the combination of remote sensing inversion and residents’ perceptions, making it difficult to directly connect research with the improvement of residents’ comfort level. More attention should be paid to residents’ perceptions and demands regarding UHI intensity and landscape cooling effects.

Xi’an is a national central city and one of China’s three major international metropolises. Because urban construction overlaps with historical sites, the urban area of Xi’an has become overly dense with buildings and has become a “furnace” city [24]. With the ultimate goal of comparing the cooling effect of urban ecological landscapes with residents’ perceptions, three detailed objectives were defined in this study: (1) to depict the spatiotemporal evolution of UHI in the central area of Xi’an City and identify the migration of the UHI center in the past 20 years; (2) to compare the cooling effects of green space and water and investigate the constraint of landscape patterns; and (3) to clarify residents’ perceptions of the landscape cooling effect on the urban thermal environment based on questionnaires and make a comparison with remote sensing inversion results. The results of this study could help in exploring feasible ways to reduce urban thermal risks in terms of rational landscape layout, and provide a scientific reference for deepening our understanding of the cooling mechanism of the ecological landscapes and promoting the optimization of thermal environment and rational urban planning of Xi’an City.

## 2. Materials and Methods

### 2.1. Study Area

**Figure 1:**
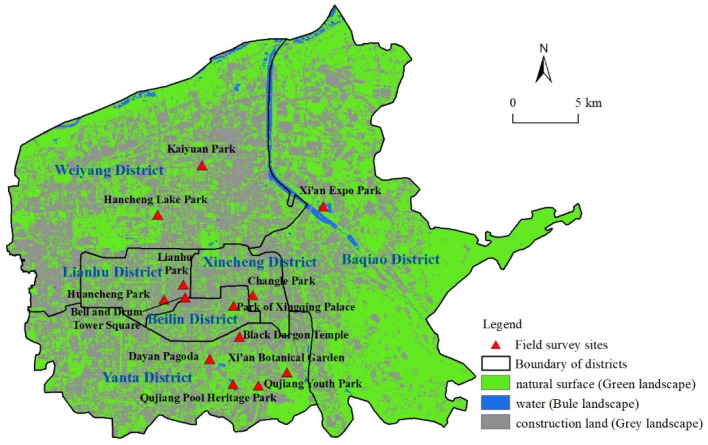
Study area and field survey sites.

Xi’an City is located in the middle of the Yellow River Basin. It has jurisdiction over 11 districts, 2 counties, 7 key development zones, and a new national district. In this paper, 6 districts (Weiyang, Baqiao, Lianhu, Xincheng, Beilin, and Yanta) were chosen as the research area, located at 34°10′–34°27′ N and 108°46′–109°17′ E. With a total territory area of 844.5 km^2^, this is Xi’an City’s core population and urban construction area (Figure 1). Among the 6 districts in the study area, Beilin, Lianhu and Xincheng Districts, located in the center, are in the old urban area, with population density of 32,383.40, 23,697.67, and 21,397.28 people/km^2^, respectively; these were the top three most populous districts in 2021, much higher than Xi’an City (1281.45 people/km^2^). The average population density of the other 3 districts is 3381.42 people/km^2^, which comprise the second circle of urban construction and population distribution in Xi’an. In 2021, the total GDP of these 6 districts accounted for 68.59% of the city, and the GDP per capita is nearly twice that of Xi’an.

Xi’an is in the warm temperate zone with a semi-humid continental monsoon climate, with annual average temperature of 13.1–14.3 °C and precipitation of 528.3–716.5 mm. High temperature is one of the major meteorological concerns in Xi’an; in 2019, the city’s national meteorological station monitored high temperature ≥35 °C 147 times in 34 days. In this paper, based on the Landsat 8 image of 13 August 2019, the land surface was divided into 3 kinds of landscapes (natural surface (green), water (blue), and construction land (grey)) by decision tree classification, which has a different mechanism for the formation of urban thermal environment. Meanwhile, residents’ perceptions were obtained through questionnaires from 13 field survey sites, as shown in Figure 1.

### 2.2. Data Sources

The datasets used in this study include remote sensing images, vector datasets and questionnaires (Table 1). Landsat images have been widely used in the study of landscape interpretation and land surface temperature retrieval for decades. Considering that the administrative district of Xi’an City spans 3 Landsat images (Path 127, Row 36; Path 127, Row 37; Path 126, Row 36), there will be time inconsistencies when splicing the images, and the value in the over-lapping area will increase the uncertainty regardless of whether the average value or the value of a certain scene is used [25]. Therefore, 6 districts in the central area of Xi’an City from the same scene (Path 127, Row 36) were selected as the research area.

The cloud-free Landsat images used in this paper were all acquired from Geospatial Data Cloud (https://www.gscloud.cn/, accessed on 12 October 2021). Four images from Landsat5 TM for 16-day intervals and Landsat8 OLI/TIRS for 16-day intervals were sampled, which were acquired on 22 August 1999, 9 August 2006, 15 August 2014 and 13 August 2019. Except for the panchromatic band and the thermal infrared band, the spatial resolution of the multispectral bands of Landsat 5 TM and Landsat 8 OLI is 30 m, which is the basic spatial resolution of landscape interpretation analysis in this study. Furthermore, the thermal infrared bands are the basis of LST retrieval, based on ENVI 5.3, preprocessing of these bands was performed, including radiometric calibration, atmospheric correction, and geometric correction. The vector datasets consisted of the administrative boundaries of Xi’an City and the field survey sites, the data of administrative boundary was obtained from the Resource and Environment Science and Data Center (https://www.resdc.cn/, accessed on 10 September 2021), and the longitude and latitude of the field survey sites were obtained by portable GPS positioning during the questionnaire surveys. The questionnaire dataset was collected from a zonal sampling survey of typical urban parks in Xi’an during a heat wave in July 2021.

### 2.3. Interpretation Landscape Types

Using remote sensing images to identify the underlying surface as the basis for LST retrieval, in this paper, the landscape types were classified by decision tree based on expert knowledge. Among them, the Blue landscape includes rivers and reservoirs; the Green landscape encompasses woodland, grassland, garden land, and cultivated land; and the Grey landscape indicates construction land, flattened unconstructed land, sandy land, and bare land. Decision tree classification does not require a priori probability distribution, and it can effectively suppress the noise of training samples and solve the problem of missing attributes [26], which is obviously superior to other remote sensing image classification methods in terms of algorithm flexibility. In this study, typical biophysical parameters were selected as the basis for classification. The Normalized Difference Vegetation Index (NDVI) was used to identify the Green landscape, the Automated Water Extraction Index (AWEI) proposed by Feyisa (2014) was chose to determine the Blue landscape [27], and the Normalized Differenced Barren Index (NDBI) was used to reflect the information of construction land and bare land [28]. The following equations were used to calculate these parameters:

(1) $$NDVI=\left(NIR-Red\right)/\left(NIR+Red\right)$$

(2) $$AWEI=4\times \left(Green-SWIR_{1}\right)-\left(0.25\times NIR+2.75\times SWIR_{2}\right)$$

(3) $$NDBI=\left(SWIR_{1}-NIR)/(SWIR_{1}+NIR\right)$$

In Equations (1)–(3), *NIR* is the near infrared reflectance, *Red* is the spectrum of the red band, *Green* is the spectrum of the green band, and *SWIR*_1_ and *SWIR*_2_ are the first and second shortwave infrared reflectance, respectively.

The decision tree used *NDVI* as the starting point for judgment, and the rules of interpretation were as follows. When a pixel satisfies *NDVI* > 0.2, it will be judged as the Green landscape [29], otherwise, proceed to the next step. Then, take *AWEI* > 0 as the criterion for whether there is water information [30], and the pixels that fail to pass go to the next step for judgement of impervious surface and bare land, and these pixels are identified as Grey landscape if they satisfy *NDBI* > 0 [31]. However, if pixels pass none of the above steps, they will be classified as an Unknown type [32]. For the Unknown pixels, the nearest Landsat images in the time series are checked. If the Unknown pixel is already classified as one of the three landscapes (Green, Blue, or Grey) in the previous or subsequent images, that specific landscape type will be assigned to it; if the pixel is recognized as Unknown among all images, it will be assigned to the landscape type with the highest proportion of the corresponding image.

### 2.4. Land Surface Temperature Retrieval

Based on the thermal infrared bands of Landsat images, including TM band 6 and TIRS band 10, the atmospheric correction method was used for LST retrieval in this study. This method is composed of three steps: radiometric calibration and atmospheric correction, emissivity calibration, and LST conversion.

Step one: Radiometric calibration and atmospheric correction. As shown in Equation (4), radiation calibration can convert the digital number (DN) value of a remote sensing image from TM band 6 and TIRS band 10 into the top atmospheric radiation intensity, for which the values of gain and offset can be found in the Landsat Product Guide. The widely used atmospheric correction formula proposed by Barsi et al. (2005) [33] was chosen to correct the top atmospheric radiation intensity to the blackbody radiation using Equation (5), which shows prominent accuracy and convenience. The relevant parameters were simulated on the Atmospheric Correction Parameter Calculator provided by NASA (http://atmcorr.gsfc.nasa.gov/, accessed on 11 November 2021).

(4) $$L_{\lambda }=DN\times Multiple+Add$$

(5) $$L_{T}=(L_{\lambda }-L_{\mu }-\tau \left(1-\varepsilon \right)L_{d})/\tau \varepsilon$$

In Equations (4) and (5), *DN* is the digitized value of spectral information, *Multiple* is the gain value, *Add* is the offset value, *L_λ_* is the top atmospheric radiation intensity, *L_T_* is the blackbody radiation, and *L_μ_* and *L_d_* are the upward and downward atmospheric radiation, respectively. *Τ* is the atmospheric transmissivity, *ε* is the surface emissivity, and the unit of *L* series parameters is W/(m^2^·sr·μm).

Step two: Emissivity calibration. Surface emissivity characterizes the closeness of the surface thermal radiation to the blackbody radiation, and *NDVI* or vegetation coverage (*P_v_*) is generally used as the conversion parameter. In this paper, we estimated the surface emissivity of water, construction land, and natural surfaces covered by vegetation using the following equations based on the three landscape types identified via decision tree classification:

(6) $$\varepsilon_{water}=0.995$$

(7) $$\varepsilon_{surface}=0.9625+0.0614P_{v}+0.0461P_{v}^{2}$$

(8) $$\varepsilon_{building}=0.9589+0.086P_{v}+0.0671P_{v}^{2}$$

In Equations (6)–(8), $\varepsilon_{water}$, $\varepsilon_{surface}$, and $\varepsilon_{building}\;$are the surface emissivity of water (Blue landscape), natural surface (Green landscape), and construction land (Grey landscape), respectively. *P_v_* was calculated as shown in Equation (9):

(9) $$P_{v}=(NDVI-NDVI_{soil})/(NDVI_{veg}-NDVI_{soil})$$

In Equation (9), $NDVI_{soil}\;$is the NDVI value of construction land or bare soil, and $NDVI_{veg}$ is the NDVI value of the vegetation coverage area. $NDVI_{veg}$ and $NDVI_{soil}$ were set as 0.7 and 0.05 as empirical values. When a certain pixel satisfies *NDVI* > 0.7, *P_v_* is set to 1, and when *NDVI* < 0.05, *P_v_* is set to 0.

Step three: LST conversion. As shown in Equation (10), the blackbody radiation can be converted into LST using the sensor calibration constants. For the Landsat5 TM dataset, *K*_1_ and *K*_2_ are 607.76 W/(m^2^·sr·μm) and 1206.56 K, respectively, and for the Landsat8 TIRS dataset, *K*_1_ and *K*_2_ are 774.89 W/(m^2^·sr·μm) and 1321.08 K, respectively.

(10) $$LST=K_{2}/\ln\left(\frac{K_{1}}{L_{T}+1}\right)$$

In Equation (10), *LST* is the land surface temperature, *K*_1_ and *K*_2_ are sensor calibration constants, and *L_T_* is blackbody radiation.

## 3. Results

### 3.1. Spatiotemporal Evolution of UHI

To achieve an accurate result of LST retrieval based on a credible interpretation of landscape types, considering the time limit of high-resolution images, the Google Earth images from August 2014 and August 2019 were chosen for validation. In this process, 300 random verification points in the study area were used to extract the landscape types between the classification results and Google Earth images. The confusion matrix shows a Kappa coefficient of 0.83 and 0.87 in 2014 and 2019 and overall accuracy of 91.50% and 93.10%, which met the application requirements. The result of landscape interpretation over the four years can be seen in Figure S1, and Table S1 shows the area and proportion of the Green, Blue, and Grey landscapes in 1999, 2006, 2014, and 2019.

**Figure 2:**
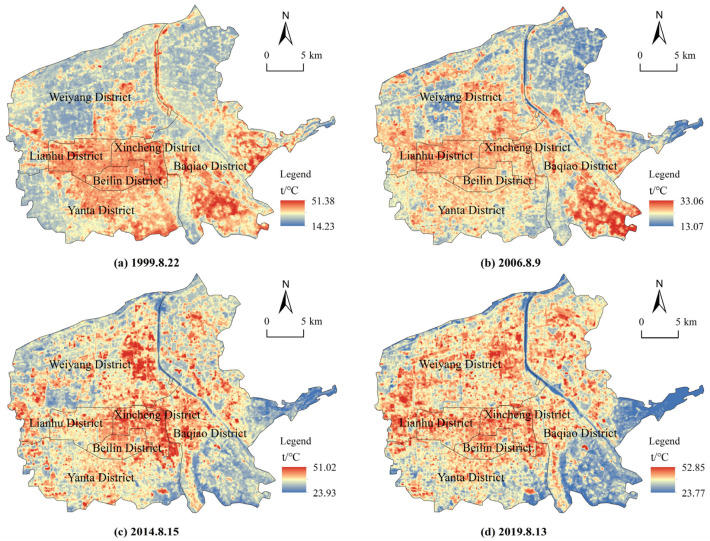
Spatial distribution of surface temperature in central Xi’an for four periods.

The LST in the six districts in Xi’an City showed significant spatial differentiation, and the high temperature area expanded northward from 1999 to 2019 (Figure 2). Specifically, on 22 August 1999, LST varied from 14.23 to 51.3 °C, with an average temperature of 30.98 °C, and the regions constructed earlier (Lianhu, Xincheng, and Beilin Districts) had higher LST. On 9 August 2006, the average LST was 23.10 °C, with extremely high temperatures in the south of Baqiao District. It is worth noting that urban construction in Weiyang and Yanta Districts accelerated significantly from 1999 to 2006, resulting in an expansion of the high temperature range. LST ranged from 23.93 to 51.02 °C and showed an average value of 33.41 °C on 15 August 2014. The UHI effect in Lianhu and Beilin Districts was reduced, and the area with extreme high temperature migrated to the east of Weiyang District. The average LST was the highest (36.66 °C) and the UHI effect was widespread on 13 August 2019. Low LST values were concentrated in urban water bodies such as the Chanhe and Bahe Rivers and the undeveloped region in the east of Baqiao District.

In order to compare the results of different years, based on the widely used mean-standard deviation method, we divided LST into five grades: high temperature area (HTA), sub-high temperature area (SHTA), moderate temperature area (MTA), sub-moderate temperature area (SMTA), and low temperature area (LTA) [34], and defined the sum of HTA and SHTA as the range of UHI [35]. Different classification thresholds were found for each year according to the mean-standard deviation method: in 1999, LTA, SMTA, MTA, SHTA, and HTA showed ranges of 14.23–26.81, 26.81–28.89, 28.89–33.07, 33.07–35.15, and 35.15–51.38 °C, respectively; in 2006, the ranges were 13.07–20.41, 20.41–21.75, 21.75–24.44, 24.44–25.79, and 25.79–33.06 °C; in 2014, the ranges were 23.93–30.82, 30.82–32.11, 32.11–34.70, 34.70–36.00, and 36.00–51.02 °C; and in 2019, the ranges were 23.77–33.78, 33.78–35.22, 35.22–38.10, 38.10–39.54, and 39.54–52.85 °C. The statistical analysis of the area proportion of LST for four periods in central Xi’an showed that the proportion of LTA decreased from 20.42 to 14.84% during 1999 to 2019, and the proportion of SMTA and SHTA were generally maintained at 15% each year. It should be noted that the area of MTA was the largest in all periods, and its proportion increased from 27.15 to 40.86%, which was mainly transformed from LTA and SMTA. The proportion of UHI composed of SHTA and HTA has always been above 30%, which is concentrated in the urban core areas with dense population, residences, and commerce.

**Figure 3:**
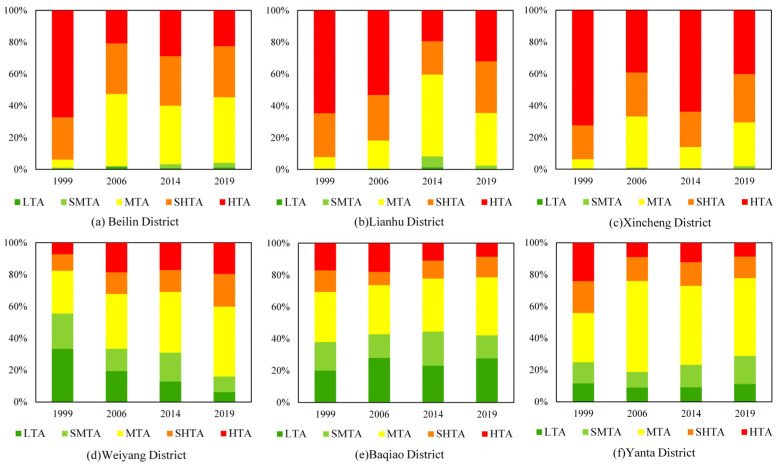
Area proportion of LST for four periods in six districts of Xi’an.

Figure 3 depicts the proportions of LST grades in the 6 districts during different periods. It can be found that the range of UHI of Beilin, Lianhu, and Xincheng Districts, which were developed earlier in the last 20 years, was significantly larger than in the other three districts. The proportion of UHI area was mostly over 50%, whereas the LTA area was less than 1%. With the outward-oriented expansion of urban construction, as well as the promulgation of the “Green space system planning of Xi’an City (2004–2020)”, the functions of the urban center have been loosened, and green infrastructure construction has been continuously strengthened. In this context, the UHI effect in Beilin, Lianhu and Xincheng Districts has been mitigated significantly and gradually transformed into MTA from 1999 to 2019. On the contrary, since the implementation of the Hundred Villages Reform to City plan in 2007, Weiyang District, located in the north of the study area, has gradually transformed from a northern suburb into a new center. However, accompanying urban thermal environment risks also became apparent. The proportion of UHI area in this district increased from 17.64 to 40.18% over the past 20 years. In contrast, the range of UHI in Yanta and Baqiao Districts decreased steadily, but the threat of continuous transfer from LTA to HTA remained.

### 3.2. Migration of the UHI Center

In this paper, hot–cold spot analysis, standard deviation ellipse, and gravity center were comprehensively applied to clarify the spatial migration of UHI in Xi’an City. Considering the resolution difference of the thermal infrared band among different sensors, the study area was divided into 360 m resolution grids, and the average LST of each grid was extracted. The standard deviation ellipse and the gravity center can effectively reflect the overall structure of geographic elements, and represent the directionality of data distribution [36]. The hot–cold spot analysis divided the samples into seven grades based on the Getis-Ord Gi* index to test whether there is a significant clustering phenomenon of high or low values: extremely cold spot (cold spot at 99% confidence), cold spot (cold spot at 95% confidence), relatively cold spot (cold spot at 90% confidence), insignificant spot, relatively hot spot (hot spot at 90% confidence), hot spot (hot spot at 95% confidence), and extremely hot spot (hot spot at 99% confidence) [37].

**Figure 4:**
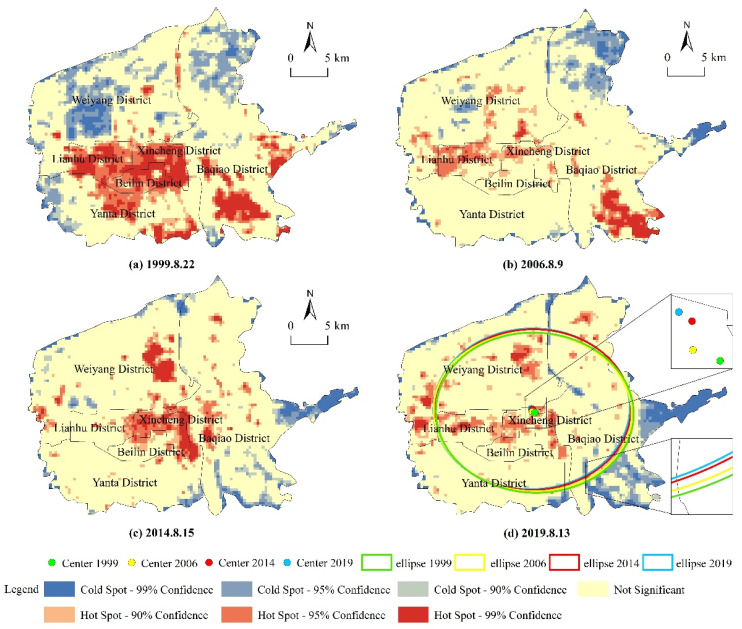
Migration of hot–cold spots, standard deviation ellipse, and gravity center of UHI.

According to the results of hot–cold spot analysis, the hot spot areas showed a trend of shrinking–transferring–diffusing during three periods (1999–2006, 2006–2014, and 2014–2019), and Weiyang District in northwest Xi’an showed an obvious trend of cold to hot in the past 20 years (Figure 4). Specifically, in the first period, the distribution of cold and hot spots was relatively stable, but the range of extreme hot and extreme cold spots shrank significantly. In the second period, most of the UHI hot spots in Baqiao and Lianhu Districts disappeared, whereas extreme hot spots appeared in the east of Weiyang District. The reason is that Weiyang District was positioned as a new administrative center in 2007 [38], and a number of projects were constructed, such as the Xi’an North Railway Station and Municipal Administrative Center. Hence, northwest Xi’an changed rapidly and became the main driving force for the migration of UHI hot spot from 2006 to 2014. In the third period, the hot spots were mostly concentrated in the northwest of the study area and the cold spots expanded in the southeast. At the same time, the range of extremely hot spots in old urban areas shrank significantly. On the one hand, this was due to the restraining effect on the thermal environment of the height limit policy in urban centers, which is aimed at maintaining the historical architectural style. On the other hand, it is related to the heat reduction effect of urban public green spaces and the gradually emerging benefits of green construction. Nonetheless, the UHI center remained relatively spatially stable during the study period. As can be seen in Figure 4d, the UHI gravity center slightly migrated within the range of 108.985°–108.990° E and 34.292°–34.296° N, always located in the north of Xincheng District. The standard deviation ellipse also shows the steadiness of the UHI center; the extent of ellipse for each year was 456.80, 453.75, 455.64 and 455.39 km^2^, respectively, reflecting the process of UHI first gathering northward and then gradually spreading.

Table 2 shows the transition of landscapes with different LST grades from 1999 to 2019. Among the landscapes that transferred out, the LTA had the largest transfer area (153.73 km^2^), half of which was converted to MTA, which was distributed all over the study area from 1999 to 2019 (Figure S2). This was followed by the HTA (138.03 km^2^), more than 70% of which converted to MTA, SMTA, and LTA. It can be seen in Figure S2 that these regions are mainly concentrated in Lianhu and Baqiao Districts. SHTA had the smallest transferred-out area (102.38 km^2^), which was mainly converted to MTA. Among the landscapes that transferred in, the area was in the order of MTA > SHTA > LTA > HTA > SMTA. The largest contribution of transferred-in area of MTA, SHTA, and HTA came from a lower LST grade. Comparing Figure 4 and Figure S2, the urban thermal environment in the old urban area of central Xi’an was obviously relieved.

### 3.3. Evaluation of Cooling Effect of Green Space and Water in Urban Parks

Based on LST in 2019, in this study, we chose 13 typical parks as samples to explore the cooling effect of green space and water, and their locations are shown in Figure 1. These samples were chosen based on a comprehensive consideration of location, passenger flow, and landscape proportion. Combining the interpretation of three landscapes (Green, Blue, and Grey) with high-resolution Google Earth images (0.5 m resolution), the ranges of green space and water landscape patches in each park were identified. In this paper, parks with more than 30% water area were classified as Water Parks (including Huancheng Park, Hancheng Lake Park, Xi’an Expo Park, and Qujiang Pool Heritage Park), whereas the rest were considered Green Parks, and the Dayan Pagoda as well as Bell and Drum Tower Square, with comparatively little green space and water landscape, were selected as the control samples. The green spaces and water landscape patches in the four water parks were evaluated compare the difference in cooling effect.

The cooling effect can be evaluated in terms of cooling intensity and cooling amplitude; the former is the maximum difference between the average temperature of a specific area outside the green space or water landscape and that area’s average temperature [16], and the latter is the range of cooling effect, which is usually expressed as the distance corresponding to the cooling intensity [17]. A buffer analysis of landscape patches in each park was performed at 30 m intervals. To quantify the cooling effect, the difference (ΔT) between mean LST of 15 buffer rings ranging from 30 to 450 m (Tn) and the mean LST within the landscape patch (T0) was calculated. The maximum value of ΔT was taken as cooling intensity, and cooling amplitude was defined as the corresponding buffer distance. The following processing was performed when setting the buffer [39]: the intersection part was deleted when the buffers of two adjacent parks overlapped, and the corresponding patch was removed to eliminate interference if there was green space or water in the buffer.

**Figure 5:**
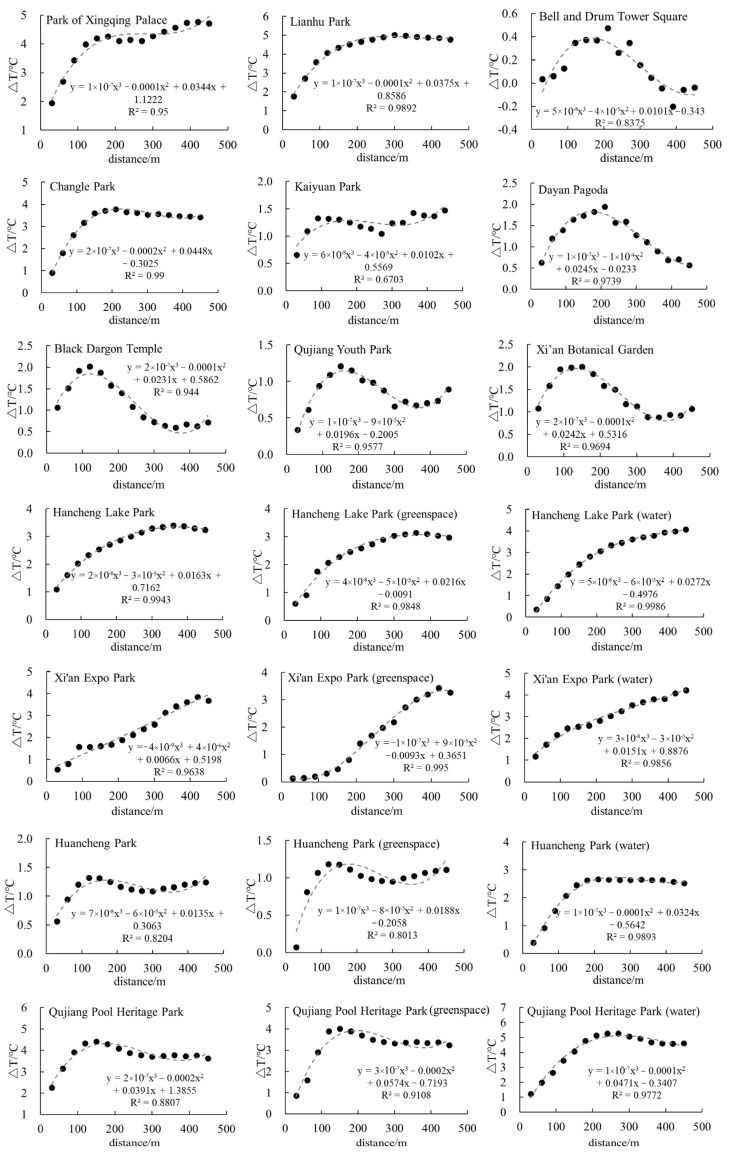
Relationship between ΔT of different buffers and distance from parks.

Figure 5 shows the variation of ΔT in multi-buffers of urban parks, demonstrating that the cubic polynomial can well reflect the significant cooling effect of parks on their surroundings, and the diverse shapes of fitting curves indicate that the cooling intensity and amplitude of urban parks differ significantly. Among them, Lianhu Park, Changle Park, Hancheng Lake Park, Hancheng Lake Park (green space), Huancheng Park (water), and Qujiang Pool Heritage Park (water) had similar fitting curves, with ΔT rising rapidly at a short distance (0–200 m) before dropping slightly beyond 400 m. The ΔT of Xi’an Expo Park and its internal green space and water patches, and Hancheng Lake Park (water) increased steadily with distance. The fitting curves of Kaiyuan Park and Park of Xingqing Palace show frequent fluctuation, with the first temperature inflection points appearing at around 200 m and 100 m, respectively. The ΔT curves of Black Dragon Temple, Qujiang Youth Park, Xi’an Botanical Garden, Huancheng Park, Huancheng Park (green space), Qujiang Pool Heritage Park, and Qujiang Pool Heritage Park (green space) displayed an increase–decrease–increase trend. ΔT often reached its peak within the range of 120–150 m from the park, and dropped rapidly after the first inflection point, the cooling effect of which was obvious, but the operating distance was short. However, the fitting curves of the control samples were significantly different from the others, showing a narrow cooling effect range and no rising trend of ΔT.

Table 3 shows that the average LST of each park ranged from 31.92 to 39.82 °C, with approximately 70% of the samples concentrated in the 33–34 °C range. Among the types of parks, the average LST of Bell and Drum Tower Square and Dayan Pagoda reached 39.82 and 37.07 °C, respectively. The control samples with the highest average LST reflected that a lack of green space and water has a direct effect on the formation of the urban thermal environment. The average inner temperature was 35.29 °C in the Green Parks, and 33.95 °C in the Water Parks. In the same park, the average inner temperature of water patches was generally more than 0.5 °C lower than that of vegetation patches, indicating that the relief effect of water on the urban thermal environment is more significant than that of green spaces; furthermore, an increased proportion of water has a positive effect of alleviating UHI as well. In terms of cooling effect (Table 3), the cooling intensity of parks ranged from 0.47 to 5.27 °C, with an average of 3.00 °C. Among the parks, Bell and Drum Tower Square had the weakest UHI mitigation ability, whereas Qujiang Pool Heritage Park had a large and concentrated area of water, which could fully act as a “cold island”. The average cooling amplitude of all samples was 241.43 m, with a maximum of more than 450 m and a minimum of 90 m. The average cooling intensity of the control samples, Green Parks and Water Parks was 1.21, 2.80 and 3.25 °C, respectively, and Water Parks had the greatest cooling amplitude (262.50 m).

## 4. Discussion

### 4.1. Correlation between Landscape Pattern and LST

In this study, Fragstats 4.2 was used to calculate the patch area (A), patch perimeter (P), and perimeter-area ratio (PARA) of each sample listed in Table 3. PARA reflects the complexity of the shape of landscape patches; the higher the value, the more complicated the shape. The result shows that the average value of index A was 68.33 ha, with Xi’an Expo Park and Lianhu Park having the highest and lowest values, respectively. The average P index was 10661.43 m, with the longest perimeter in Huancheng Park and the shortest in Lianhu Park. The average PARA was 210.63; the most complex patch could be found in Huancheng Park (water) (PARA = 677.19), and the shape of Xi’an Expo Park is relatively the most regular.

**Figure 6:**
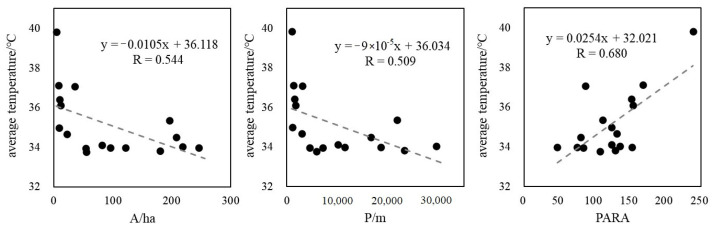
Relationship between average temperature and landscape pattern metrics of typical urban parks.

Understanding the relationship between UHI and landscape pattern can provide important support for urban green space system planning. The relationship between average LST and landscape pattern metrics of each park in 2019 was determined in this paper. As explained in Section 3.3, the internal temperature of water is significantly lower than that of green space, which belongs to a special landscape patch in parks. In order to avoid the influence of special samples, four single patches of water were excluded. Figure 6 shows that the average temperature inside urban parks was significantly negatively correlated with landscape area and perimeter (*p* < 0.05), implying that the cooling effect of parks would be significantly enhanced as the area increased, which is consistent with the results of previous studies [39]. Moreover, the Pearson correlation coefficient revealed that LST was more sensitive to changes in area than perimeter, indicating that expanding the park area would be more effective than increasing the perimeter to reduce UHI. PARA was significantly positively correlated with the average temperature of parks (*p* < 0.01), indicating that more complex shapes are often accompanied by higher LST, which has been confirmed in many studies [40,41]. In general, expanding the area of urban parks and controlling the complexity of their shape would help to reduce UHI. However, due to differences in land prices, the growth of planned areas of green infrastructure is limited. Hence, more attention should be paid to maximizing the cooling effect by increasing the proportion of water, increasing the vegetation density and optimizing the shape of parks.

### 4.2. Residents’ Perceptions of the Cooling Effect of Green Space and Water

Exploring urban residents’ perceptions could effectively support urban planning for improved quality of human settlements. In this study, a questionnaire was designed on urban residents’ perceptions of high temperature and the cooling effect of the ecological landscape. Respondents who were randomly selected at 13 urban parks filled out the questionnaire independently. In total, 360 questionnaires were distributed, and 325 valid ones were returned. The questionnaire included the respondents’ basic information, daily life and work situation, perception of high temperature weather, and perception of the cooling effect of green space and water bodies, etc. (see Supplementary S2 for the complete questionnaire). According to the results, 92.31% of the respondents reported that UHI had an impact on them, and 41.85% stressed that the impact was large or great. Further, 81.23% of the respondents reported that their daily life and work situations were disturbed, and 44.92% said they sought medical treatment due to high temperature, and women were more vulnerable to heat stress.

The analysis of urban residents’ perception of the cooling effect on ecological landscapes included scoring, judging trend, and expressing perceptions of the impact of landscape area or shape on the cooling effect. In this study, the respondents were asked to rate the cooling effect of green space and water surrounding their living environment in 1999, 2006, 2014, and 2019 on a scale from 0 to 5 (0 means no feeling, 5 means obvious feeling), so as to compare with the result of remote sensing inversion. After guiding the respondents to recall their feelings as much as possible, and after removing incompletely scored samples, the results showed that the scores were generally higher for green spaces than water; the multi-year average scores were 2.74 and 2.60, respectively. Additionally, the scores for the cooling effect of green space (1.90–3.72) and water (1.79–3.60) showed an increasing trend. However, studies have shown that the cooling amplitude and intensity of water make it more beneficial than green space of the same area [9]. Therefore, the residents’ perceptions were biased because the scale of water bodies in Xi’an City is still relatively limited, and their cooling capacity has not been fully utilized.

Regarding the judgment of the trend of the cooling effect of green space and water, 73.23% of the respondents noted a gradually improving trend, only 6.15% had the opposite opinion, and the rest could not make a clear judgment. The chi-squared test revealed significant differences in judgment among respondents of various ages and educational levels with various kinds of home cooling equipment (Table 4). Among them, elderly residents over 60 years old had the most polarized views on this issue, with the highest proportions believing that the cooling effect had improved (26.47%) or deteriorated (41.79%). Young people aged 18–29 (41.79%) comprised the major group who thought that the trend was unclear. The importance of better education to raise awareness of extreme weather events and perception of the thermal environment has been highlighted in previous research [42,43]. In this study, residents with a bachelor’s degree tended to be more optimistic and uncertain about the cooling trend, whereas those with a lower level of education were more inclined to have a pessimistic attitude, which indicates that community education and publicizing relevant knowledge should be strengthened. Furthermore, people who had three types of cooling equipment at home accounted for the highest proportion of all judgments.

Regarding the perception of the influence of landscape pattern on the cooling effect, 79.08% of respondents thought that areas of green space and water would have an influence. However, their understanding of the constraints of landscape shape was limited, and responses of “affects”, “does not affect”, and “unsure” accounted for 40.92, 38.46, and 20.62%, respectively, highlighting the importance of incorporating a correlation analysis between landscape pattern and UHI into urban construction planning. As shown in Table 5 and Table 6, gender, education, occupation, and income were significant factors influencing residents’ perception of the constraint of landscape area on the cooling effect, and different occupations and housing types had a significant influence on respondents’ judgments on the constraints of landscape shape. Among them, men (50.19%), people with an undergraduate education (36.19%), retirees (28.40%), and people with a monthly income less than CNY 2000 (30.65%) who believed that area would affect the cooling effect accounted for the highest proportions. However, the number of respondents who disagreed with this decreased as their educational level increased, which reflected that higher levels of education are often accompanied by more objective cognition and greater sensitivity to environmental changes [44]. In addition, students (30.83%) and respondents who did not live on the top floor of their apartment building (67.67%) identified strongly with the concept that landscape shape has an influence on the cooling effect, and retirees had the highest proportion of disapproving or uncertain attitude.

### 4.3. Comparison between Remote Sensing Inversion and Residents’ Perceptions

In this paper, residents’ perceptions were compared with the spatiotemporal distribution of UHI and the cooling effect of urban parks, so as to comprehensively demonstrate their mutual relationship. On the one hand, the results show high consistency between remote sensing inversion and residents’ perception. It was found that the proportions of HTA and SHTA in Beilin, Lianhu, Xincheng, Baqiao, and Yanta Districts showed a decreasing trend, which was directly reflected by the optimistic attitude of most respondents to the cooling effect of urban parks. At the same time, more than 70% of respondents noted that the cooling effect of the urban ecological landscape was gradually increasing, which was consistent with the annual decrease in HTA from 1999 to 2019 obtained by remote sensing inversion. This positive perception to the cooling trend indicates that they have confidence that UHI will be reduced in the context of current urban and ecological construction in Xi’an City.

On the other hand, residents’ perceptions also deviated from the actual effects of the ecological landscape on UHI to a certain extent. The evaluation of the cooling effect revealed that water has better cooling ability, whereas residents gave higher scores to green space. In addition, the significant influence of park areas on the cooling effect was confirmed by the residents’ perceptions; most respondents demonstrated strong recognition of this issue, but their awareness of the constraints of park shape was still insufficient. This reflects the need to strengthen the research on the combination of remote sensing inversion and residents’ perceptions. It is also urgent to promote the publicity and popularization of urban thermal risk as well as the cooling effect of the ecological landscape among communities. In this way, the cognition degree of residents and their adaptability to UHI could be gradually improved.

### 4.4. Limitations and Future Research Directions

Exploring effective cooling methods for UHI is unavoidable for the coordinated development of urban construction and ecological environment. This study combined multi-temporal analysis based on remote sensing with urban residents’ perceptions to strengthen their mutual support and demonstration, and highlighted urban residents’ intuitive demand for UHI mitigation, which would be helpful to connect the research results with the urban planning and construction of Xi’an City. However, several aspects of this study could be improved.

First, in terms of the selection of datasets and samples, the continuity of LST inversion data in this paper was relatively deficient due to the difficulty of obtaining clear remote sensing images at the same time every year. Multi-source data could be integrated in further research to carry out day-night and seasonal tracking analyses, and refined data sources such as SPOT and Quick Bird could be used to improve the accuracy of identifying landscape patches. Furthermore, due to labor constraints during the questionnaire survey process, the samples only covered 13 typical parks in Xi’an City; more comprehensive results could be obtained in further research by expanding the survey scale. In addition, in order to ensure that the sample would show obvious characteristics and have enough passenger flow during the investigation, some newly built urban parks were chosen in this study, which limited the continuous monitoring of the cooling effect from 1999 to 2019. To explore the long-term evolution of the cooling effect, continuous monitoring of independent samples should be considered in future study.

Second, in terms of the mechanism analysis and generalization of conclusions, this paper mainly relied on mathematical statistics to interpret the cooling effect of parks on UHI. Systematic analysis and model simulation of landscape thermodynamic processes were not included, and the interpretation of the internal mechanism of the correlation between landscape pattern and LST needs to be strengthened. In addition, although the findings of this study may reflect the cooling characteristics of typical parks in Xi’an City, it remains to be verified whether the relevant conclusions are applicable to other areas, and whether the constraints of the area, perimeter, and shape of landscape patches on the cooling effect of parks can be verified by outdoor field observations, which will require more extensive interdisciplinary and collaborative research in the future.

## 5. Conclusions

In this study we investigated the spatiotemporal evolution of UHI in the six districts of Xi’an City from 1999 to 2019 using multi-source data and various methods, and evaluated the cooling intensity and cooling amplitude of green space and water in typical parks. In addition, the influence of landscape pattern on the cooling effect was investigated, and residents’ perceptions were compared with remote sensing inversion based on a questionnaire survey. The results show that the LTA was concentrated in Weiyang and Baqiao Districts, and its area was shrinking. The HTA expanded northward over the six districts, whereas the MTA covered the largest area and is still expanding. The extent of UHI in Beilin, Lianhu, and Xincheng Districts, which were developed earlier, was significantly larger than the other three districts. With the outward expansion of urban construction and the release of central functions, the UHI effect has gradually eased in the old urban area. During the periods 1999–2006, 2006–2014, and 2014–2019, the hot spots of UHI generally migrated to the northwest of Xi’an City, and showed a trend of shrinking–transferring–diffusing.

The average LST inside the urban parks showed a gradient of Water Parks < Green Parks < control samples, with water patches having a greater cooling capacity than green spaces. Among the samples, the average cooling intensity and cooling amplitude in 2019 were 3.00 °C and 241.43 m, respectively, and the cooling effect was strengthened with an increased proportion of water in parks. In addition, because the internal LST of parks is more sensitive to changes in area than perimeter, green spaces and water landscapes with more regular shape would be more useful in reducing UHI. During the process of urban construction, it is not feasible to control LST by reducing infrastructure over a large area. Therefore, to support the regulation of the urban thermal environment as well as the process of urban planning and construction, it is necessary to control the complexity of ecological patches in urban parks and increase the proportion of water.

The questionnaire showed that 92.31% of respondents believed that the high temperature weather in Xi’an has an impact on them. Nearly half of them reported having received medical treatment due to high temperature, and the proportion of those experiencing symptoms was higher among women than men. Furthermore, 73.23% of the respondents thought that the cooling effect of green space and water has improved in recent years, whereas those with a lower education level showed less confidence in this regard. Furthermore, 79.08% and 40.92% of the residents thought that the area and shape, respectively, of landscape patches would have an influence on their cooling effect. Among those two groups, the former was mainly made up of men, undergraduates, retirees, and those with a monthly income of less than CNY 2000, and the latter was mainly students and people who did not live on the top floor of their apartment building.

## Figures and Tables

**Table 1:** Information of datasets.

Data Type	Dataset	Temporal Resolution	Spatial Resolution	Acquisition Date
Remote sensing images	Landsat 5 TM images	16-day	30 m for bands 1–5 and 7; 120 m for band 6	22 August 1999, 9 August 2006
	Landsat8 OLI/TIRS images	16-day	30 m for bands 1–7 and 9; 15 m for band 8; 100 m for bands 10–11	15 August 2014, 13 August 2019
Vector datasets	Administrative boundaries of Xi’an City	/	/	2015
	Field survey sites	/	/	July 2021
Questionnaires	325 valid questionnaires obtained from investigation	/	/	July 2021

**Table 2:** Transition probability matrix of LST grades in Xi’an during 1999–2019 (km^2^).

Type	LTA	SMTA	MTA	SHTA	HTA	Total
LTA	/	23.51	79.86	27.26	23.10	153.73
SMTA	16.91	/	64.30	22.80	17.46	121.46
MTA	33.54	30.64	/	38.16	29.57	131.90
SHTA	20.21	14.28	51.99	/	15.90	102.38
HTA	35.96	15.78	51.60	34.69	/	138.03
Total	106.61	84.21	247.75	122.92	86.02	/

**Table 3:** Average inner temperature, cooling intensity (°C), and cooling amplitude (m) of typical urban parks.

Name of Park	Inner Temperature	Cooling Intensity	Cooling Amplitude	Name of Park	Inner Temperature	Cooling Intensity	Cooling Amplitude
Park of Xingqing Palace	33.95	4.25	180	Xi’an Expo Park	33.98	3.86	420
Huancheng Park	34.03	1.32	120	Xi’an Expo Park (green space)	34.49	3.43	420
Huancheng Park (green space)	35.36	1.18	120	Xi’an Expo Park (water)	33.58	4.22	450
Huancheng Park (water)	33.61	2.67	210	Dayan Pagoda	37.07	1.95	210
Lianhu Park	34.98	5.03	300	Black Dragon Temple	37.12	2.02	120
Bell and Drum Tower Square	39.82	0.47	210	Qujiang Pool Heritage Park	33.97	4.41	150
Changle Park	34.67	3.78	210	Qujiang Pool Heritage Park (green space)	34.11	4.02	150
Hancheng Lake Park	33.83	3.40	360	Qujiang Pool Heritage Park (water)	31.92	5.27	240
Hancheng Lake Park (green space)	33.98	3.14	360	Qujiang Youth Park	36.11	1.21	150
Hancheng Lake Park (water)	33.43	4.06	450	Xi’an Botanical Garden	33.77	2.00	150
Kaiyuan Park	36.41	1.32	90				

**Table 4:** Differences in judgments of the trend of cooling effect for different groups of people.

Category	Type	Getting Better (n (%))	Unsureness (n (%))	Getting Worse (n (%))	χ^2^	*p*
Age	<18	21 (8.82%)	5 (7.46%)	1 (5.00%)	23.054	0.011
	18–29	57 (23.95%)	28 (41.79%)	1 (5.00%)		
	30–39	25 (10.50%)	8 (11.94%)	3 (15.00%)		
	40–49	33 (13.87%)	10 (14.93%)	2 (10.00%)		
	50–59	39 (16.39%)	6 (8.96%)	2 (10.00%)		
	≥60	63 (26.47%)	10 (14.93%)	11 (55.00%)		
Education	Junior high school and below	50 (21.01%)	7 (10.45%)	7 (35.00%)	16.790	0.032
	High school (secondary specialized school or vocational-technical college)	66 (27.73%)	11 (16.42%)	6 (30.00%)		
	Junior college	38 (15.97%)	14 (20.90%)	3 (15.00%)		
	Undergraduate	76 (31.93%)	29 (43.28%)	3 (15.00%)		
	Postgraduate and above	8 (3.36%)	6 (8.96%)	1 (5.00%)		
Cooling equipment at home	None	1 (0.42%)	0	1 (5.00%)	16.113	0.013
	One	68 (28.57%)	27 (40.30%)	2 (10.00%)		
	Two	40 (16.81%)	12 (17.91%)	2 (10.00%)		
	Three	129 (54.20%)	28 (41.79%)	15 (75.00%)		

**Table 5:** Test on the influence of area on cooling effect for different groups of people.

Category	Type	Area Influences Cooling Effect (n (%))	Area Does not Influence Cooling Effect (n (%))	Unsure (n (%))	χ^2^	*p*
Gender	Male	129 (50.19%)	17 (56.67%)	28 (73.68%)	7.473	0.024
	Female	128 (49.81%)	13 (43.33%)	10 (26.32%)		
Education	Junior high school and below	44 (17.12%)	13 (43.33%)	7 (18.42%)	23.767	0.003
	High school (secondary specialized school or vocational-technical college)	70 (27.24%)	8 (26.67%)	5 (13.16%)		
	Junior college	41 (15.95%)	4 (13.33%)	10 (26.32%)		
	Undergraduate	93 (36.19%)	3 (10.00%)	12 (31.58%)		
	Postgraduate and above	9 (3.50%)	2 (6.67%)	4 (10.53%)		
Occupation	Public institution	18 (7.00%)	0	6 (15.79%)	30.045	0.018
	Company employee	17 (6.61%)	1 (3.33%)	5 (13.16%)		
	Service industry	14 (5.45%)	1 (3.33%)	1 (2.63%)		
	Student	66 (25.68%)	2 (6.67%)	7 (18.42%)		
	Outdoor worker	21 (8.17%)	3 (10.00%)	5 (13.16%)		
	Retiree	73 (28.40%)	9 (30.00%)	6 (15.79%)		
	Individual business	8 (3.11%)	3 (10.00%)	1 (2.63%)		
	Unemployed	15 (5.84%)	5 (16.67%)	5 (13.16%)		
	Other	25 (9.73%)	6 (20.00%)	2 (5.26%)		
Income	Under CNY 2000	57 (30.65%)	6 (28.57%)	8 (33.33%)	15.620	0.048
	CNY 2000–4000	51 (27.42%)	9 (42.86%)	2 (8.33%)		
	CNY 4000–6000	44 (23.66%)	2 (9.52%)	3 (12.50%)		
	CNY 6000–8000	19 (10.22%)	2 (9.52%)	6 (25.00%)		
	Over CNY 8000	15 (8.06%)	2 (9.52%)	5 (20.83%)		

**Table 6:** Test on the influence of shape on cooling effect for different groups of people.

Category	Type	Shape Influences Cooling Effect (n (%))	Shape Does not Influence Cooling Effect (n (%))	Unsure (n (%))	χ^2^	*p*
Occupation	Public institutions	7 (5.26%)	9 (7.20%)	8 (11.94%)	26.452	0.048
	Company employee	6 (4.51%)	9 (7.20%)	8 (11.94%)		
	Service industry	9 (6.77%)	6 (4.80%)	1 (1.49%)		
	Student	41 (30.83%)	19 (15.20%)	15 (22.39%)		
	Outdoor worker	11 (8.27%)	13 (10.40%)	5 (7.46%)		
	Retiree	31 (23.31%)	41 (32.80%)	16 (23.88%)		
	Individual business	4 (3.01%)	7 (5.60%)	1 (1.49%)		
	Unemployed	9 (6.77%)	13 (10.40%)	3 (4.48%)		
	Other	15 (11.28%)	8 (6.40%)	10 (14.93%)		
Housing types	Not top floor of apartment building	90 (67.67%)	99 (79.20%)	53 (79.10%)	16.500	0.036
	Top floor of apartment building	20 (15.04%)	17 (13.60%)	2 (2.99%)		
	Flat building	16 (12.03%)	9 (7.20%)	8 (11.94%)		
	Villa	3 (2.26%)	0	1 (1.49%)		
	Other	4 (3.01%)	0	3 (4.48%)		

## Data Availability

The data presented in this study are available on request from the corresponding author.

## Supplementary Materials

The following supporting information can be downloaded at: https://www.mdpi.com/article/10.3390/ijerph192214880/s1, Figure S1: Landscape types over four years in the study area; Figure S2: Spatial distribution of LST grades over four periods in central Xi’an; Table S1: Area and proportion of landscape types over four years in the study area; Supplementary S2: Questionnaire of urban residents’ perceptions of high temperature and cooling effect of ecological landscape.
