# Supplementary material for: Cooling Effect of Green Space and Water on Urban Heat Island and the Perception of Residents: A Case Study of Xi’an City
Source: Int J Environ Res Public Health. 2022 Nov 12;19(22):14880. doi: 10.3390/ijerph192214880 (PMC9690120; doi:10.3390/ijerph192214880)
Supplement: Supplementary file 1 [file ijerph-19-14880-s001.zip › ijerph-1951742-supplementary.pdf]

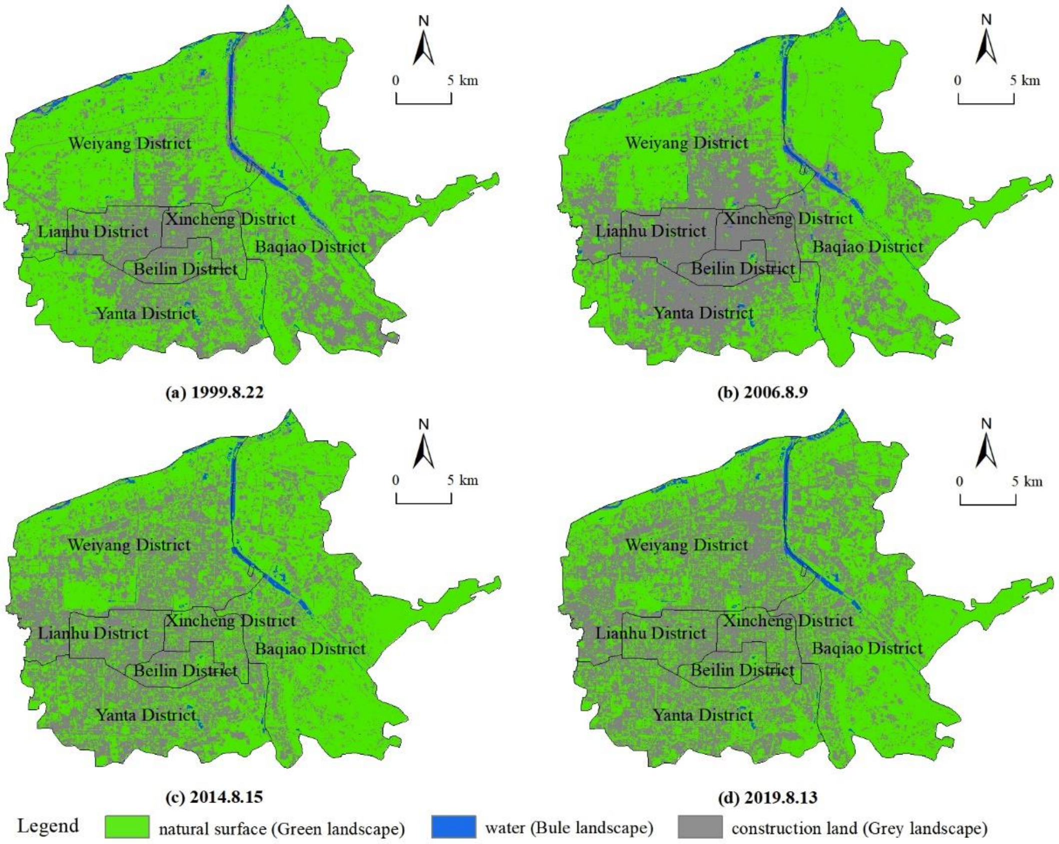

Figure S1. Landscape types over four years in the study area.

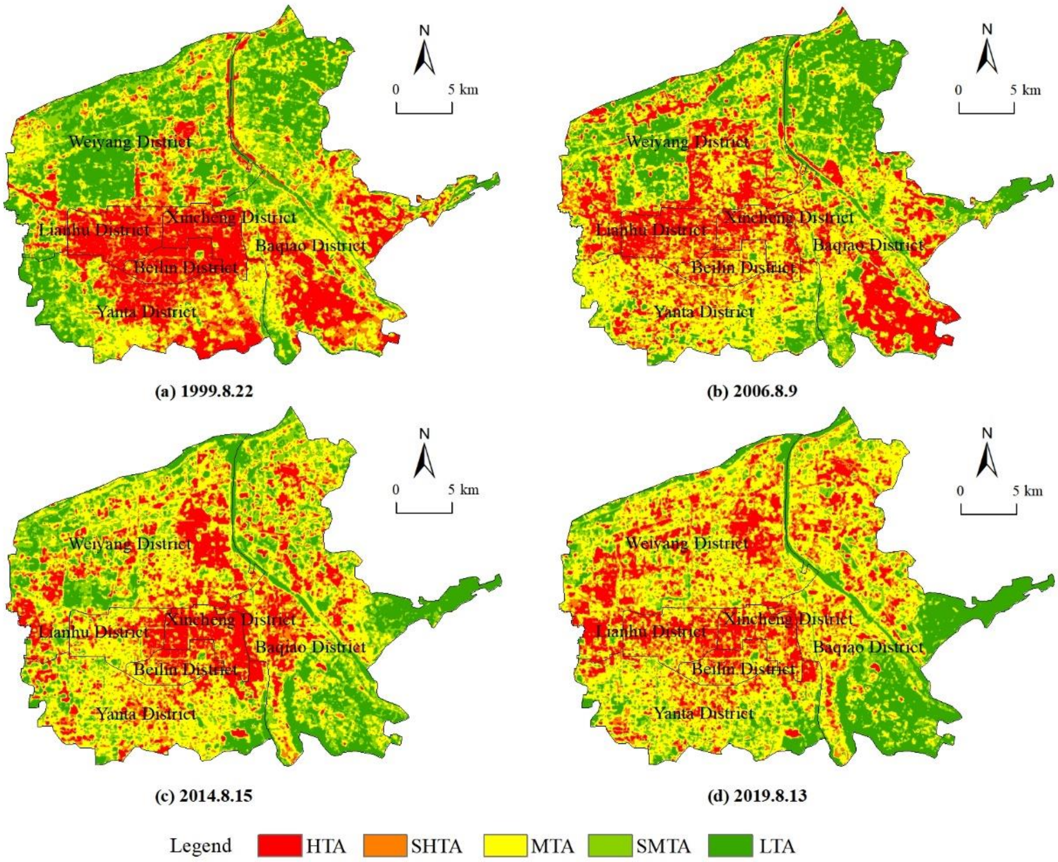

Figure S2. Spatial distribution of LST grades over four periods in central Xi’an.

Table S1. Area and proportion of landscape types over four years in the study area.

Year	Green landscape		Blue landscape		Grey landscape	
	Area/km^2	Proportion/%	Area/km^2	Proportion/%	Area/km^2	Proportion/%
1999	576.45	68.26	8.63	1.02	259.43	30.72
2006	516.75	61.19	8.73	1.03	319.03	37.78
2014	512.30	60.66	11.02	1.31	321.17	38.03
2019	465.68	55.14	12.38	1.47	366.44	43.39

Supplementary S2. Questionnaire of urban residents' perceptions of high temperature and the cooling effect of ecological landscape

Dear Residents:

Hello! We are students from School of Geography and Tourism, Shaanxi Normal University. We are studying on the cooling effect of the ecological landscape on urban heat island in the central area of Xi'an City, we look forward to provide suggestions for the prevention of thermal environmental risks in Xi'an City through our study. Your answer will provide a valuable data basis for our study. Sincerely thanks for your cooperation and support.

Instructions:

1. The survey is anonymous, and all the data would be strictly confidential and only used for research, which would not cause any inconvenience to you;
2. Please read the questions and options carefully, choose the answer that best matches your actual situation, please check the options or fill in directly;
3. Only one answer should be selected for all the questions in the questionnaire, except for the questions marked with "multiple choice".

Date: ______Year ______Month ______Day Name of investigator: ____________

Investigation site: _______District _______Street (town) _____ Community (village)______

Part 1: Basic Information

**A1. Gender**	1. Male 2. Female
**A2. Age**	
**A3. Physical condition**	1.Healthy 2. Well 3. General 4. Have disease: ________
**A4. Education**	1.Junior high school and below 2.High school (secondary specialized school or vocational-technical college) 3.Junior college 4.Undergraduate 5.Postgraduate and above
**A5. Occupation**	1.Public institutions 2.Company employee 3.Service industry 4.Student 5.Outdoor worker 6.Retiree 7.Individual business 8.Unemployed 9.Others: ______
**A6. Monthly income**	1.Under 2000 CNY 2.2000~4000 CNY 3.4000~6000 CNY 4.6000~8000 CNY 5.Over 8000 CNY
**A7. Residence time in Xi'an**	1.Local people 2. Within six months 3. Six months to two years 4.Three to five years 5. Five to ten years 6.More than ten years

Part 2: Daily life

B1. What is your housing type?

1.Non-top floor of apartment building 2.Top floor of apartment building 3.Flat building 4.Villa 5.Others: _________

B2. What kind of cooling equipment do you have in your home (multiple choices)?

1.Fan 2. Air conditioner 3. Refrigerator 4. None

B3. Which of the following environments do you mostly work in?

1. Outside in the sun 2. Outdoor shade

3. Indoor without air conditioner 4. Indoor with air conditioner

B4. During hot weather, what cooling equipment do you use at work (multiple choices)?

1.Fan 2. Air conditioner 3. Refrigerator 4. None

B5. How long do you normally need to work outside in a day (single choice)?

1. Within 1 hour 2. 1~2 hours 3. 2~4 hours 4. 4~6 hours 5. 6~8 hours 6. More than 8 hours

B6. How do you usually go to work or go out during the summer (multiple choice)?

1.Walk 2. Bicycle/Electric bicycle 3.Bus 4. Subway

5. Private car 6.Taxi 7.Others: _______

Part 3: Perception of high temperature weathers

C1. What degree do you think is the high temperature weather? Above_______°C

C2. How long do you think the hot weather in Xi'an usually lasts?

During______(Month) to______(Month)

C3. How do you feel the high temperature weathers affects your daily life?

1. Great 2. Large 3. General 4. Minor 5. Barely

C4. What kind of impact will high temperature weathers bring to you (multiple choice)?

1. Affect mood (psychological irritability, anxiety) 2. Travel inconvenience

3. Decrease in travel activities 4. Cost of living increases 5. Decrease in study/work efficiency

6. Cause physical discomfort 7. No influence

C5. Have you ever gone to the hospital for medical treatment due to high temperature weathers (multiple choices)?

1. Respiratory discomfort 2. Sleep disorders 3. Cardiovascular and cerebrovascular complications

4. Digestive system diseases 5. Sunburn 6. Sunstroke 7. Others: _______ 8. None

Part 4: Perception of the cooling effect of green space and water

D1. Scores for the cooling effect (On a scale of 0 to 5, 0 means no feeling, 5 means obvious feeling)?

Year	1999	2006	2014	2019
Cooling effect of surrounding green spaces				
Cooling effect of surrounding water area				

D2. In recent years, do you think the cooling effect of ecological landscape is gradually improved with the construction of green space and water in Xi'an City?

1. The cooling effect is getting better 2. Not sure 3. The cooling effect is getting worse

D3. Do you think the area of water or green space will have an impact on its cooling effect?

1. Yes 2. No 3. Not sure

D4. Do you think the shape of water or green space will have an impact on its cooling effect? (If not, skip question D5)

1. Yes 2. No 3. Not sure

D5. Which shape of water or green space do you think has the best cooling effect? (A score of 0 is the worst and a score of 5 is the best)

Water or greenspace landscape in a shape of roundness: _____score

Water or greenspace landscape in a shape of rectangle: _____score

Water or greenspace landscape in a shape of foursquare: _____score
Water or greenspace landscape in irregular shape: _____score

D6. Compared with greenspace and water, which one do you think has a better cooling effect?

1. Water landscape 2. Greenspace 3. Not sure

Part 5: Personal needs and suggestions

E1. What policies and measures do you want the government to take to deal with high temperature weathers (multiple choices)?

1. Guarantee water supply and power supply in hot weather 2. Provide high temperature subsidies to needy families 3. Provide heatstroke prevention medicines 4. Increase open spaces with air conditioner 5. Increase green infrastructure 6. Replenish border trees 7. Announce high temperature warnings in time 8. Adjust summer working hours 9. Others:_______

E2. Facing the high temperature weathers, do you think it is necessary to build urban greenspace and water landscape?

1. Very necessary 2. Necessary 3. General 4. Not quite necessary 5. No need at all

E3. Do you have any suggestions for the construction of water and greenspaces around your living and working environment?

____

____

____
